# Phylogenetic Affiliation of SSU rRNA Genes Generated by Massively Parallel Sequencing: New Insights into the Freshwater Protist Diversity

**DOI:** 10.1371/journal.pone.0058950

**Published:** 2013-03-14

**Authors:** Najwa Taib, Jean-François Mangot, Isabelle Domaizon, Gisèle Bronner, Didier Debroas

**Affiliations:** 1 Clermont Université, Université Blaise-Pascal, Laboratoire "Microorganismes: Génome et Environnement", BP 10448, Clermont-Ferrand, France; 2 CNRS, UMR 6023, LMGE, Aubiere, France; 3 INRA, UMR 42 CARRTEL, Thonon les Bains, France; 4 Université de Savoie, UMR 42 CARRTEL, Le Bourget du Lac, France; University of Illinois at Chicago, United States of America

## Abstract

Recent advances in next-generation sequencing (NGS) technologies spur progress in determining the microbial diversity in various ecosystems by highlighting, for example, the rare biosphere. Currently, high-throughput pyrotag sequencing of PCR-amplified SSU rRNA gene regions is mainly used to characterize bacterial and archaeal communities, and rarely to characterize protist communities. In addition, although taxonomic assessment through phylogeny is considered as the most robust approach, similarity and probabilistic approaches remain the most commonly used for taxonomic affiliation. In a first part of this work, a tree-based method was compared with different approaches of taxonomic affiliation (BLAST and RDP) of 18S rRNA gene sequences and was shown to be the most accurate for near full-length sequences and for 400 bp amplicons, with the exception of amplicons covering the V5-V6 region. Secondly, the applicability of this method was tested by running a full scale test using an original pyrosequencing dataset of 18S rRNA genes of small lacustrine protists (0.2–5 µm) from eight freshwater ecosystems. Our results revealed that i) fewer than 5% of the operational taxonomic units (OTUs) identified through clustering and phylogenetic affiliation had been previously detected in lakes, based on comparison to sequence in public databases; ii) the sequencing depth provided by the NGS coupled with a phylogenetic approach allowed to shed light on clades of freshwater protists rarely or never detected with classical molecular ecology approaches; and iii) phylogenetic methods are more robust in describing the structuring of under-studied or highly divergent populations. More precisely, new putative clades belonging to Mamiellophyceae, Foraminifera, Dictyochophyceae and Euglenida were detected. Beyond the study of protists, these results illustrate that the tree-based approach for NGS based diversity characterization allows an in-depth description of microbial communities including taxonomic profiling, community structuring and the description of clades of any microorganisms (protists, Bacteria and Archaea).

## Introduction

The development of molecular ecology was prompted by indisputable evidence that, for most environments on Earth, the majority of existing organisms had not yet been cultured. This evidence came from the analysis of sequences recovered directly from environmental samples. Vast new lineages of microbial life were uncovered by this approach, changing our picture of the microbial world and yielding a phylogenetic description of community membership [1], [2]. More precisely, the sequencing of the small sub-unit (SSU) rRNA genes highlighted new monophyletic groups or clades in the environment, such as SAR11 [3] or MGI [4] among the Bacteria and Archaea respectively. Similarly, several new lineages of protists have been discovered in oceanic systems during the last decade [5]. Recent studies conducted in lakes have also highlighted numerous phylogenetic groups, especially putative parasites (Fungi and Perkinsozoa), and this finding is modifying our view of the microbial loop and therefore, the functioning of aquatic ecosystems [6], [7].

Recent advances in next-generation sequencing (NGS) technologies are spurring progress in determining the microbial diversity of various ecosystems by highlighting, for example, the rare biosphere and the activity of these low abundance organisms [8], [9]. Currently, the pyrosequencing of amplified SSU rRNA gene variable regions is mainly used to determine bacterial and archaeal diversity and structure in various ecosystems, such as soil [10], ocean [11] or gut microbiota [12]. The recent results obtained regarding the composition and structure of the microeukaryote communities using high-throughput amplicon sequencing performed with the Roche 454 pyrosequencing platform in freshwater systems [13], [14] have fuelled the current debates on the biogeography of these microorganisms and on the role of the rare biosphere. The taxonomic assignment of such data is often inferred from supervised classification with the Ribosomal Database Project Classifier (RDP) [15], sequence similarity with BLAST [16]–[18] or both [19], [20]. Pairwise identity scores via BLAST remain the most commonly used tool for large eukaryotic datasets [14], [21]–[26]. However, as claimed by Bik et al. [26], assigning accurate taxonomy to eukaryotic operational taxonomic units (OTUs) is more difficult than the approaches used for Bacteria; the relative paucity of sequences in public eukaryotic databases results in many sequences without significant top BLAST matches [26]. Furthermore, the best BLAST match assigns a single organism as the most likely phylogenetic neighbor, without specifying the level of relatedness (class, order or phylum) of the compared sequences [27].

Phylogenetic methods assess relatedness among various groups of sequences by inserting unknown OTU sequences within a known phylogeny. On the one hand, these methods allow query sequences to be affiliated with their relatives. Tree-based assignment is, therefore in theory, a more robust approach [28] and current FLX Titanium longer reads now make it possible to extract phylogenetic information with a high degree of reliability [29]. On the other hand, phylogenetic analyses allow for the description of clades, which may lead to new insights into the structure and functioning of ecosystems, as previously mentioned. Moreover, these phylogenetic analyses are not limited to the taxonomic assignment of an individual sequence as implemented in bioinformatic pipelines dedicated to NGS and used in microbial ecology studies (mainly on 16S rRNA gene amplicons): phylogenies can also be used to compare environments (beta-diversity) using methods based on tree topology and/or branch length such as the popular tool UNIFRAC (unique fraction metric) [30]. Although more robust, these methods are less frequently used than BLAST or probabilistic classifiers, as they require more computing resources (Table S1). Though large computational capacity is now more accessible (e.g., QIIME [20] can be implemented on a cloud), massively parallel sequencing projects that seek to elucidate the phylogenetic structure of microbial populations are still faced with the attendant computational challenges of classifying the sequences obtained.

In this work, we introduce a tree-based treatment designed for analyzing massively parallel sequencing outputs that automatically affiliates sequences from SSU rRNA gene amplicons and builds phylogenetic trees composed of very large numbers of sequences. As short-read sequence data (e.g., 100 base sequences generated by the Illumina sequencing platform) provide limited phylogenetic resolution [29], our work is focused on the treatment of moderately long (∼ 450 bp obtained for example with Titanium platforms) to near full-length sequences. Designed for the analysis of any microorganism (protists, Bacteria and Archaea), the value of this treatment is highlighted here on the protist diversity as the pipelines dedicated to the study of eukaryotic pyrotags are still scarce. Indeed, 16S rRNA gene reads were widely investigated in previous studies [31]–[33] to assess bacterial diversity, which enhanced the development of specific 16S rRNA gene analytical tools. However, 18S rRNA gene surveys and tools allowing for the accurate and rapid taxonomic affiliation of protists from NGS data are needed because the number of studies dealing with protists diversity is currently increasing (e.g., [14], [34]). We first tested the accuracy and speed of phylogenetic affiliation on large fragments of well-annotated 18S rRNA gene sequences (>1,200 bp) and on short sequences that simulate pyrosequencing outputs. Secondly, the different methods of taxonomic assignment (i.e., tree-based, similarity and probabilistic approaches) were compared with each other, in a first attempt to determine the best method for affiliating protists in the context of massively parallel sequencing of amplicons. Thirdly, the accuracy of phylogenetic affiliation was compared on amplicons covering different variable regions (V1 to V9), and finally, a dataset of original pyrosequencing data obtained from lacustrine small protists was analyzed by the tree-based approach that was developed.

## Results

### Evaluation of performance on reference sequences

**Figure 1:**
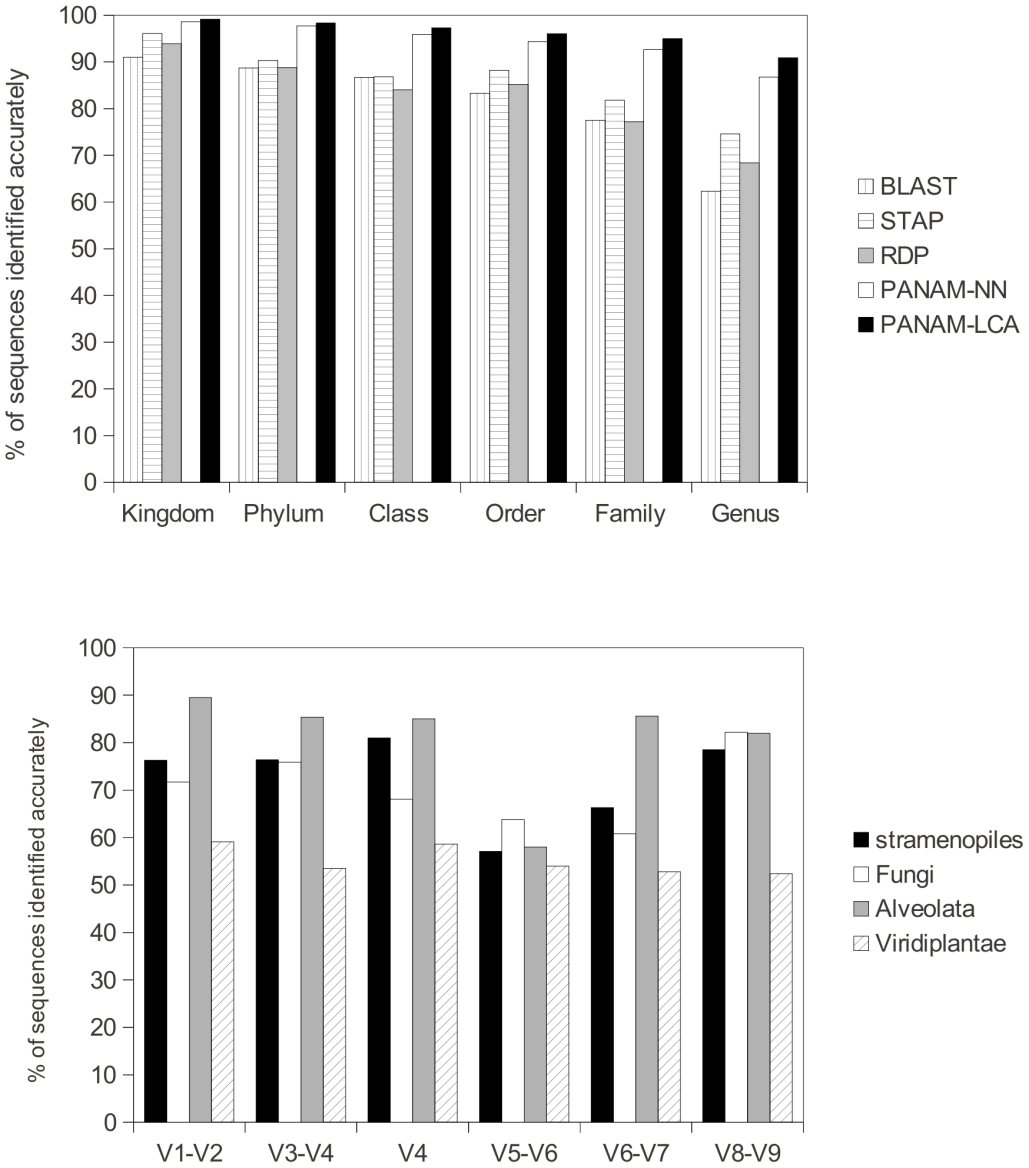
Accuracy of the phylogenetic affiliation of PANAM compared to different approaches and on different regions . 1.A. Accuracy of the phylogenetic affiliation of PANAM-LCA, PANAM-NN, STAP, BLAST and RDP Classifier. 1,000 near-full-length sequences were randomly picked from the reference database and removed from it for the simulations. For PANAM, simulations were repeated 5 times and the standard variation is less than 0.03. 1.B. Accuracy of the phylogenetic affiliation in relation with the variable region targeted. The specificity was tested with PANAM-LCA and a sequence length equal to 400 bp.

In the analysis of near full-length reference sequences of 18S rRNA gene, taxonomic groups were found in similar proportions to those initially present in the samples. Our phylogenetic affiliation method, referred to as PANAM (Phylogenetic Analysis of Next-generation AMplicons), was more accurate using LCA (lowest common ancestor) assignment for the different taxonomic ranks, ranging from 99.1% to 90.8% versus 98.6% to 86.7% for PANAM using the NN (nearest neighbor) method (Figure 1.A). For comparison, when refining affiliations from kingdom to genus, the accuracy of the standard phylogenetic affiliation using ClustalW [35] and PHYML [36] as implemented in STAP, ranged between 96.1% and 74.6%. At the finest phylogenetic level studied (i.e., genus), BLAST and RDP allowed for the affiliation of 62.3% and 68.4% of reference sequences. Thus, our phylogenetic affiliation method outperformed the other methods on near full-length sequences. However, as environmental sequences are generally quite divergent from referenced ones and their affiliation needs to be checked manually, sequences belonging to freshwater clades [6], [7] were also processed by our phylogenetic affiliation method to evaluate how it behaved on these datasets. The phylogenetic analysis of these environmental sequences (Sanger, >1,200 bp) enabled us to retrieve the affiliations obtained by other authors together with the delineation of freshwater clades corresponding to Cercozoa clade [6] and Perkinsea clades 1 and 2 [7] (Figure S1).

Different 18S rRNA gene regions were targeted by simulating amplicons with lengths of 200 and 400 bp starting from a conserved region given by the following forward primers: NSF4, NSF370, NSF573 NSF963, NSF1179 and NSF1419 (Table S2). Because the V8–V9 region is often missing in public databases, the results obtained from this region were based only on 300 sequences included in the reference database. The affiliation results at the genus rank differed according to length, variability within the studied region and method used for taxonomic affiliation (Table 1). For the six regions tested, the accuracy increased with amplicon length for both affiliation methods implemented in PANAM, LCA and NN. Considering the affiliation methods, LCA specificity was higher than that of NN for fragments of 200 bp only for the V1 and V8 regions, and LCA specificity was always better for fragments of 400 bp. The comparisons with the other affiliation tools implemented in pipelines dedicated to pyrosequencing results showed that at 200 bp, BLAST outperformed RDP, STAP and PANAM, with the exception of the V8 region, for which PANAM (LCA) gave the highest result (68.7%). In contrast, for 400 bp amplicons, the most accurate affiliations were obtained with PANAM, with the exception of the V5–V6 amplicon. In this last region, we observed a decrease in the accuracy of the affiliation, coupled with a sharper decline for the phylogeny-based affiliations. The specificity therefore varied between 64.2% (V5–V6) and 79.2% (V8–V9) at the genus level.

**Table 1:** The specificity percentage values at the genus level for BLAST, RDP, STAP and PANAM (NN and LCA).

Starting position	Region	Length	BLAST	RDP	STAP	PANAM-NN	PANAM-LCA
NSF4	V1	200 bp	69.3	59.4	58.2	60.7	63.1
	V1–V2	400 bp	73.2	62.9	72	73.3	78.1
NSF370	V3	200 bp	61.7	54	54.2	55.9	50.2
	V3–V4	400 bp	70.9	67	70.8	70.2	73.3
NSF573	V4	200 bp	70.3	65.5	66.8	62.5	55.5
	V4	400 bp	72.3	67.8	69.9	74.6	76.8
NSF963	V5	200 bp	57.7	54.4	49.9	51.5	41.8
	V5–V6	400 bp	68.8	65.1	65.2	60.6	64.2
NSF1179	V6	200 bp	66.7	62.8	59.1	53.5	52.4
	V6–V7	400 bp	71.0	68.8	69.7	71.9	74.3
NSF1419	V8	200 bp	68.5	66.7	62.9	62.8	68.7
	V8–V9	400 bp	74.4	69.3	72.4	74	79.2

The specificity corresponds to the number of genus correctly affiliated among the detected ones, computed from forward primers for 200 bp and 400 bp amplicons. These values correspond to the mean computed from five samples of 1000 sequences (with the exception of V9 region computed with 300 sequences). The standard variation is less than 0.05.

In addition to the accuracy of assignment, this phylogenetic affiliation method was developed to optimize processing time for large datasets. Thus with a 2 GHz Intel(R) Xeon(R) and 24 GB RAM and with a single 32-bit CPU, PANAM can process the phylogenetic analysis of 1000 eukaryotic OTUs of 400 bp in approximately 20 minutes, regardless of the affiliation method. The run time increased with the number of OTUs, regardless of the length. For example, for 400 bp, the run time ranged from 24 minutes for 5000 OTUs to 6 days and 14 hours for 1 M eukaryotic OTUs. For near full-length sequences, PANAM was able to process 1 M sequences in 16 days (Figure S2).

### Reliability of the phylogenetic affiliation in relation to the region targeted and the taxa of interest

The reliability of affiliations was compared for 400 bp reads spanning the 18 S rRNA gene for four taxonomic groups: Alveolata, Stramenopiles, Fungi and Viridiplantae at the genus level (Figure 1.B). Generally, the fragment affiliation depended on the taxonomic group and the region considered. According to previous results, the regions from V5 to V6 gave, on average, the weakest accuracy. Another general trend observed in this analysis was a poor taxonomic restitution for sequences belonging to Viridiplantae compared to other groups, between 52.4% and 59.1% regardless of the region targeted. The best specificity values for Stramenopiles, Alveolata and Fungi were obtained in different regions: V1–V2 (89.5%), V4 (81%), and V8–V9 (82.2%) respectively. The taxonomic affiliation for these three groups from the V8–V9 region was relatively similar, from 78.5% to 82.2%.

### Tree-based analysis of pyrosequencing data from small lacustrine protists

*In silico* simulations have shown that primers NSF573 and NSR1147, used to target the V4 region of the 18S rRNA gene captured the greatest diversity (data not shown) and that the region amplified by these primers is suitable for taxonomic affiliation (Table 1). The reads were clustered at 95% similarity, and 6% of the OTUs (4% of reads) defined from this pyrosequencing run matched with Metazoa sequences and were not processed further. The diversity and richness indexes obtained for each environment are shown in Table 2. The lowest and highest richness indexes (Chao1) were found on Anterne Lake and Villerest Lake respectively, whereas the normalized indexes (based on 3759 sequences) showed that Bourget Lake harboured the largest number of species (Table 2). This normalization also had an effect on the richness estimates in Godivelle Lake and Geneva Lake.

**Table 2:** Main characteristics of the lakes studied and richness and diversity indexes of small protists inferred from the pyrosequencing of amplicons .

Main characteristics			Richness and diversity						Richness and diversity normalized					
Lakes	Trophic status	Coordinates	Sequences	OTUs	Chao1	Shannon	ACE	Coverage	Sequences	OTUs	Chao1	Shannon	ACE	Coverage
Anterne	ultraoligotrophic	45°59'28''N, 6°47'54''E	17092	150	282.1	1.7	292.8	99.6	3759	51	93.0	0.5	121.3	99.3
Aydat	eutrophic	45°39′50″N, 2°59′04″E	8574	239	328.5	2.5	319.1	99.1	3759	176	235.1	2.59	237.6	98.5
Bourget	mesotrophic	45°43′55″N, 5°52′ 06″E	3759	294	442.6	4.0	478.6	96.7	3759	295	436.2	3.95	469.5	96.7
Geneva	mesotrophic	46°27'52''N, 6°33'31''E	10045	345	442.4	4.2	462.4	99.0	3759	158	199.0	3.70	203.5	98.9
Godivelle	ultraoligotrophic	45° 23′ 04″ N, 2° 55′ 25″ E	8742	234	317.8	3.8	313.2	99.2	3759	229	371.8	4.02	340.3	97.7
Pavin	oligomesotrophic	45°29′45″N, 2° 53′ 18″ E	11618	254	389.0	3.5	364.8	99.2	3759	157	287.7	3.39	244.7	98.4
Sep	oligomesotrophic	46° 02′ 51″ N, 3° 02′ 47″ E	7795	309	406.1	3.9	418.6	98.8	3759	232	329.5	3.79	322.4	98.0
Villerest	hypereutrophic	45° 59' 36'' N, 4° 2' 12'' E	8427	369	482.3	4.2	472.5	98.7	3759	277	399.5	4.14	373.9	97.4

**Figure 2:**
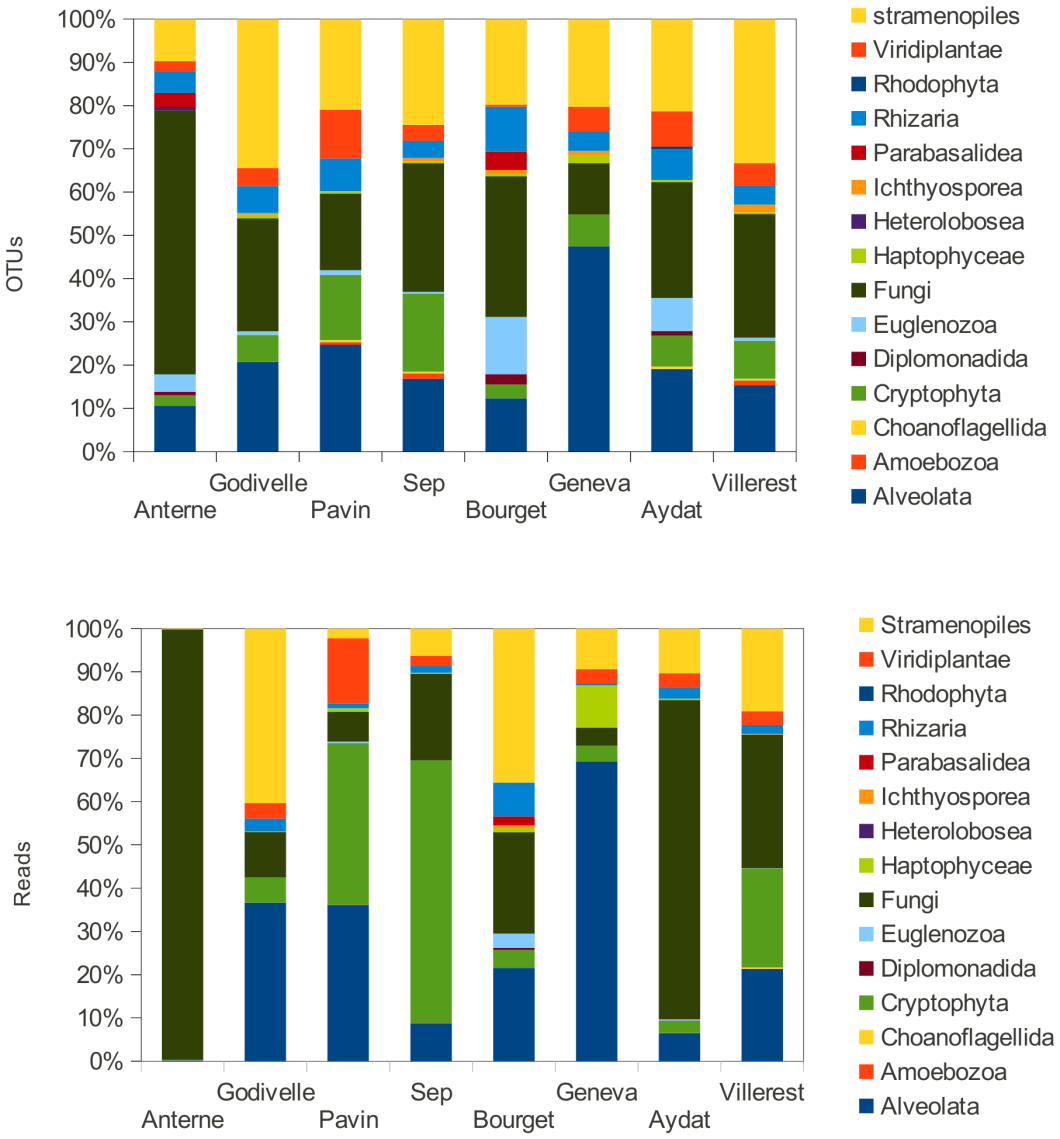
Proportions of the main phyla detected in the 8 lakes studied. The proportions are computed in term of OTUs (top) and reads (bottom) (see Table S3).

**Figure 3:**
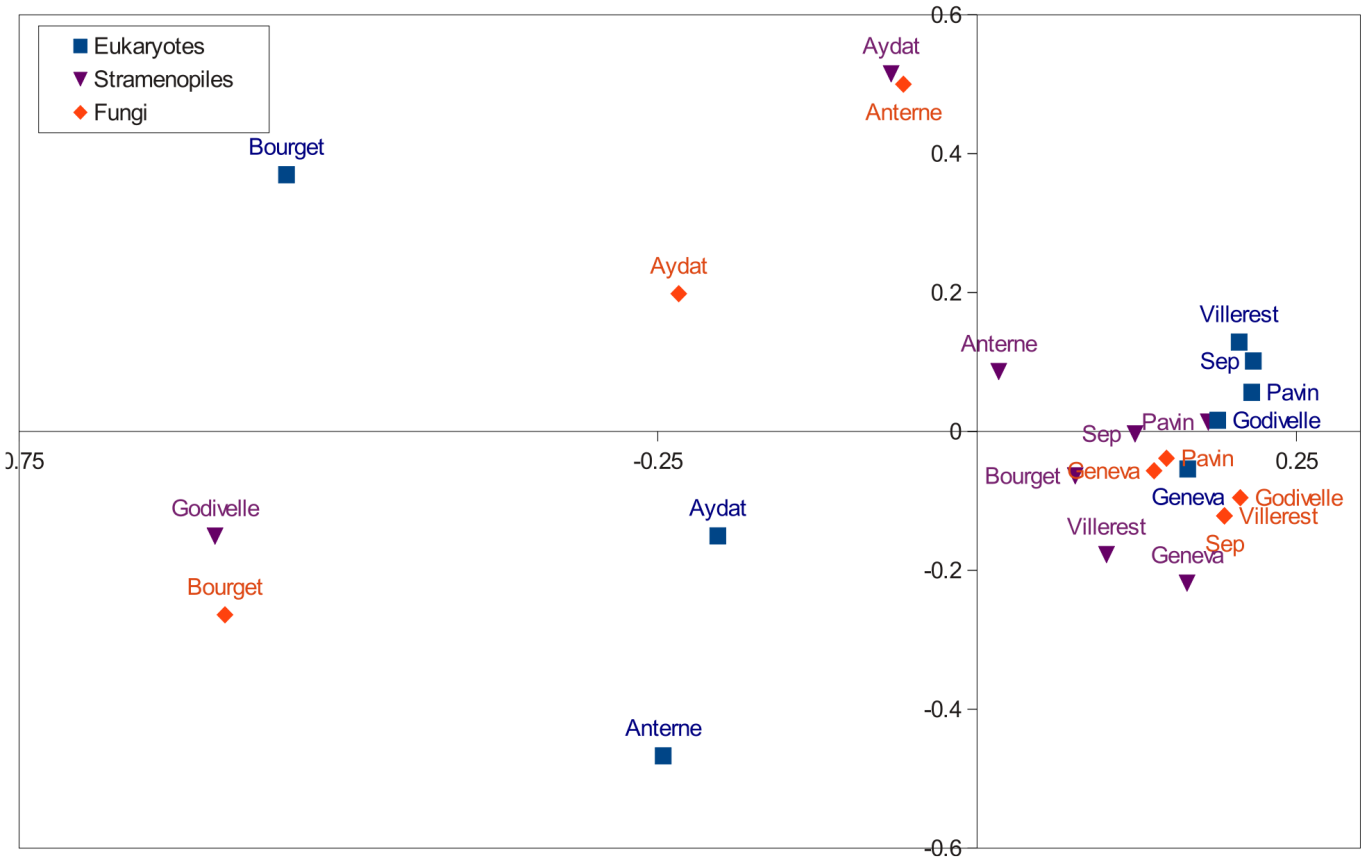
Principal coordinate analysis computed using a Unifrac distance metric from the phylogenies of the Stramenopiles, Fungi and the total eukaryotes. This analysis permit to differentiate environments according to their taxonomic composition. For example, Lake Godivelle seems to be different from the other lakes for the Stramenopiles, while it is similar for all eukaryotes.

In the lakes studied, regarding level 2 and 3 from EMBL taxonomy (displayed in Table S3, a PANAM table output, including number of sequences, OTUs and diversity indexes), the major phylogenetic groups were Fungi, Alveolata and Stramenopiles representing 73.2% of OTUs and 78.6% of sequences (Figure 2). These mean values mask some disparities between lakes. Thus, Anterne Lake harboured mainly reads affiliated to Fungi (99.4% of total), whereas the main phylum in Geneva Lake was Alveolata (Figure 2; Table S3). Sequences belonging to the phylum Cryptophyta were the most abundant in Pavin Lake and Sep Lake.The results highlighted the presence of freshwater clades delineated in previous studies [6], [37] such as Cryptophyta_2 to Cryptophyta_4, Rhizophydium or Cryptomycota (previously known as LKM11) among Fungi (Table S3). Sequences derived from Fungi, which were very abundant in sequence libraries from Anterne Lake and Aydat Lake, belonged to this last Cryptomycota clade (Table S3, Figure S3). These data demonstrate the presence of Chlorophyta and Haptophyta in all of the lakes studied, with the exception of Anterne Lake, which is characterised by an over-representation of Fungi and an absence of Haptophyta. This tree-based approach allows for the study of beta-diversity from phylogenies. The UNIFRAC metric showed that Bourget, Aydat and Anterne Lakes differed from other ecosystems regardless of the phylogenetic level (total Eukaryotes, Stramenopiles and Fungi) at which the analysis was performed (Figure 3).

**Figure 4:**
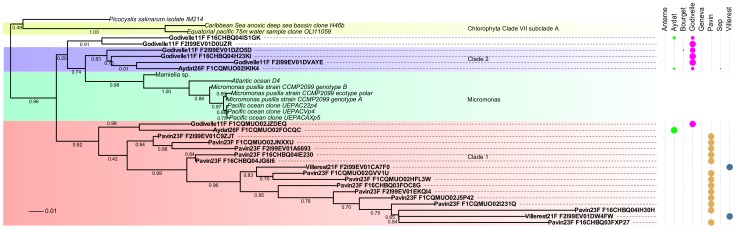
Main putative clades detected among Mamiellophyceae (Chlorophyceae) based on 18S SSU reads (425 bp ± 114). The OTUs affiliated to Chlorophyceae were generated at 95% similarity. A profile alignment was processed using HMMalign and the phylogeny was built by FASTTREE2 with 100 bootstraps. The distribution of the OTUs among different lakes shows a main presence of clade 1 in Lake Pavin while clade 2 is mainly present in Lake Godivelle.

In a comparison of the OTUs found in this study to those present in previous studies on the small protists, only 4.8% were previously detected in lakes. If only the dominant OTUs (>1% of reads) are taken into account, then the proportion of OTUs similar to specific lacustrine sequences increased to 19.7%. Moreover, new light is shed on putative clades of small protists. Specifically, these clades include the chlorophycean group of Mamiellophyceae, represented in Figure 4; Foraminifera (Rhizaria); Dictyochophyceae (Stramenopiles); and Euglenida (Euglenozoa). These clades were supported by high bootstrap values (> 0.8), included 23, 14, 17 and 23 OTUs respectively, and were found in at least three of the eight lakes. The novel clade within the Euglenozoa was composed only of OTUs present at less than 1% of reads.

## Discussion

As the interplay between evolution and ecology receives more attention in ecosystem studies [38], there is greater interest in phylogenetic approaches for deciphering the mechanisms that govern the diversity and functioning of communities and ecosystems. However, the phylogenetic methods that are typically applied to Sanger-sequenced SSU rRNA are computationally expensive and cannot be readily used to handle NGS datasets; therefore, pyrosequencing reads are mainly analyzed by other approaches. The method described in this study is a response to the challenge of analyzing hundreds of thousands of SSU rRNA genes in a phylogenetic framework, inferring taxonomies from sister sequences and describing clades. This method has been implemented and tested for microorganisms with an emphasis on protists, which are not well served by bioinformatics tools dedicated to NGS data, although the early focus on bacterial and archaeal diversity has recently broadened to include eukaryotic microorganisms [39], [40]; thus, the database provided in PANAM includes reference sequences from protists, Bacteria and Archaea and can be used for taxonomic assignment of all microorganisms.

### Accuracy of affiliation methods for protist sequences

Our taxonomic affiliations were compared with BLAST, a tool commonly used for the identification of microorganisms especially microeukaryotes (e.g., [22]); RDP, which is currently used to classify bacterial and archaeal SSU rRNA sequences and fungal LSU rRNA sequences; and STAP implemented in WATERS [41]. This method, based on ClustalW alignments and PHYML phylogenies, is a standard method for taxonomic affiliations based on phylogenetic analyses. The RDP Classifier [42] is often considered to be restricted to bacterial and archaeal taxa [26] and therefore, is not used for eukaryotic classification of SSU rRNA genes after amplicon pyrosequencing. We used this tool for the first time for taxonomic affiliation of 18S rRNA gene amplicons generated with high-throughput pyrotag sequencing. The affiliation of simulated amplicons were obtained by the RDP Classifier trained on the near full-length sequences of the reference database used in PANAM. Surprisingly, trimming the reference database to the primer region did not result in an improvement of classification for 18S rRNA gene sequences (data not shown), in contrast to the results of Werner et al. [43] on 16S rRNA gene sequences. As noted by these authors, a naïve Bayesian classification depends on the training set size. The weak performance on the truncated sequences could thus be explained by the limited number of 18S rRNA gene sequences in public databases compared with 16S rRNA gene sequences, particularly for the V9 region (see the discussion below).

The comparison of the tree-based method proposed with these tools in the context of taxonomic affiliation of 18S rRNA gene amplicons shows that regardless of the method that is used, taxonomic reliability depends on the sequence length and amplicon location on the SSU rRNA gene sequence. These results, which to our knowledge have not been examined for 18S rRNA gene sequences, are consistent with observations of 16S rRNA gene sequences from Bacteria and Archaea [44].

Our results mostly illustrate the impact of sequence length on phylogenetic methods, which appears to be the main limitation of this approach. According to Liu et al. [31], it is possible to use short fragments from the 16S rRNA gene to draw the same conclusions as with full-length sequences. However, by comparing different affiliation methods, they also noted that the short reads generated by pyrosequencing (i.e., 200 bp) were likely to be problematic for inferring phylogeny due to their small number of bases; similarity and probabilistic methods are therefore the most accurate. However, our analysis, similar to the one proposed by Jeraldo et al. [29] for 16S rRNA gene sequences, demonstrates that with the current average length achieved by the pyrosequencers (Titanium generation; > 400 bp), phylogenetic methods are reliable and offer an advantage over other methods such as RDP. From 400 bp amplicons, the phylogenetic affiliation method implemented in PANAM outperforms the classical tools dedicated to NGS analysis at the genus level with the exception of amplicons sequences covering the V5–V6 region of the SSU rRNA gene. Phylogenetic methods are generally considered superior to other approaches for taxonomic affiliation [45] as they assess relatedness between a set of sequences. They are also considered to be difficult to automate as i) their reliability greatly depends on the quality of the alignments, which need to be validated by experts in the field, and ii) they use intensive, time-consuming methods for tree building.

In this study, we use the curated alignments sequences provided by SILVA, which is, at least for eukaryotic sequences, the only up-to-date curated database. All high-quality and near full-length aligned sequences suitable for in-depth phylogenetic analysis were selected. However, the guide-tree for eukaryotes provided by SILVA, in contrast to the other domains, represents only an approximate phylogeny. Tree-based approaches can implement other tools based on the tree-insertion methods like pplacer [46] as proposed by Bik et al. [28]. Similarly to STAP, this tool analyzes one sequence at a time. Thus, clades may be, at best, approximated from a frozen backbone tree, while the addition of distant taxa, as can be expected from environmental sequences, may require a re-evaluation of the phylogenetic tree [46]. In terms of processing time, we demonstrated that the tree-based method described here can process 1 M sequences in a reasonable (about three hours) time scale. For comparison, while pplacer processes 10,000 sequences in ∼0.5 hour, PANAM can process 30,000 sequences in the same amount of time with the same computational resources. However, while a pyrosequencing run can produce up to 1.2 M reads, the raw sequences first go through a quality control stage that eliminates poor quality reads and replicates. Additionally, in diversity studies, the raw sequences are first cleaned (i.e., quality trimmed) and clustered, and phylogenetic analyses are applied to the representatives of each OTU and not to all of the raw reads from a run. Consequently, in current studies of diversity, the effective number of sequences to be affiliated is on the order of tens of thousands, which can be processed in a few hours on a personal computer.

### Accuracy of the protist affiliation in relation to the region targeted

The primers used for the taxonomic assignment of Bacteria traditionally span the regions V3, V6 and V9 of the SSU rRNA gene [12], [47]. However, some studies [32], [48] suggest that the V6 region is not optimal for taxonomic affiliation as it overestimates richness and the number of OTUs at different cut-offs [49]. In the microeukaryotic field, the regions V2–V3 [13], V3 [14], [34], V4 [22], [23], [39] and V9 [21], [22], [24], [25], [39] were investigated with limited *in silico* analysis. Behnke et al. [39] partially addressed this concern because they compared the V4 and V9 regions for analyzing sequencing errors; V4 amplicons are likely more prone to an increased frequency of Roche 454 pyrosequencing homopolymer errors relative to the V9 region [22]. However, the inclusion of at least some part of the variable regions of the SSU RNA gene is necessary for the methods to retrieve sufficient signal for taxonomic affiliation. Liu et al. [32] stressed that tree-based methods are more sensitive to the 16S rRNA gene region targeted than are similarity-based methods because of different rates of evolution among regions [44], and/or the difference of homopolymer incidence and length between the regions [48]. The same conclusions can be drawn from our results from 18S rRNA gene amplicon sequences, because the accuracy of the phylogenetic affiliation for the region V5–V6 dropped for both phylogenetic methods used in this study (STAP and PANAM). Interestingly, the accuracy of the taxonomic affiliation of the main phyla varied with the region analyzed, but regardless of the variable region analyzed, simulated amplicons from Viridiplantae were always difficult to affiliate reliably at the genus level. Thus, the bias observed between variable regions [22] could be due to primers that may not anneal uniformly to all groups, but also to the bioinformatic process used for the taxonomic identification. In summary, with the exception of Viridiplantae, the V8–V9 region appears to be a good candidate for the study of protist diversity because the reliability of the taxonomic affiliation did not differ according to the phyla considered (i.e., Stramenopiles, Fungi, Alveolata). However, sequence databases such as GenBank contain many fewer sequences that include the V9 region than other variable regions.

### New insights into the small protist composition of the lacustrine ecosystem

In this analysis, our goal was not to explain the spatial pattern of the protist community composition (PCC) but to characterize the structure of these communities (richness, diversity and composition) by high-throughput SSU rRNA gene amplicon sequencing and sequence affiliation utilizing a tree-based method. We focused on the optimization of processing environmental data and on the description of the general picture of protists diversity obtained for these lakes.

For an in-depth analysis of this PCC from lacustrine ecosystems, we introduced environmental sequences and taxonomies in the reference database to delineate specific clades as defined in previous publications (e.g., [6], [37], [50]). The introduction of “environmental reference” sequences reflecting the taxonomies of protists originating from specific environments can enhance the affiliation of poorly represented environmental sequences. Phylogenetic methods provide a clear edge in describing under-studied and complex communities. However, as with other methods, the precision of sequence mapping falls off when experimental sequences lie distant from reference SSU rRNA gene sequences [51]. This observation is particularly true for environmental sequences, for which the availability of close relatives and well-annotated sequences in reference databases is limited, as is the case for the V9 region. If the referenced trees do not include known relatives branching close to experimental reads, divergent lineages form long-branch taxa with no close reference sequences at relatively deep internal nodes. This phenomenon results in a less precise taxonomic affiliation of these sequences; however, clades of interest could still be drawn, as very similar sequences (i.e., sequences with low pairwise distance) are very well preserved among tree searches from *de novo* phylogenies [29].

Most eukaryotic species are defined on morphological differences, however, as the majority of existing microorganisms on Earth have not yet been cultured, their phenotypic traits can hardly be described. Thus, environmental microbial species are delineated according to a sequence similarity cut-off based on comparisons of SSU rRNA gene sequences to demarcate operational taxonomic units [52]. Although they do not technically represent species, OTUs composed of multiple sequences can be used to describe novel species, using the provisional designation of “Candidatus”, when the SSU rRNA gene sequences are sufficiently different from those of recognized species [53]. In this study, after dataset cleaning and sorting, the reads left for the affiliation were clustered at a 95% identity threshold as proposed by Caron et al. [54] to delineate eukaryotic taxa. These authors defined this similarity threshold after studying the distribution of intra- and inter-specific variations of the 18S rRNA gene in protistan communities. However, as they pointed, this cut-off is a conservative estimator of species richness, and may mask considerable physiological diversity in some OTUs. In other studies, taxon clustering is performed at sequence similarity from 90% to 100% [23]. As the error rate of many NGS platforms in any case is ∼1% it is recommanded to cluster at a lower threshold than 99%. Some authors chose a similarity of 97% because this value is commonly used to define OTUs in Bacteria (e.g., [22]). However, this value has been defined for delineating a species from the full-length 16S rRNA gene. Thus, from *in silico* analysis of 16S rRNA genes, Kim et al. [33] showed that the clustering threshold must be chosen according to the variable region amplified and the domain studied (i.e., Archaea or Bacteria). A less conservative cut-off could overestimate the richness and diversity because in some phyla, such as diatoms, the level of intragenomic polymorphism in the SSU rRNA gene can reach 2% [55]. Finally, in a previous study, Mangot et al. [56] defined a threshold of 95 % by adding an internal standard (a clonal sequence derived from a copy of the 18S rRNA gene in Blastocystis subtype 4 genome) before amplifying and sequencing the DNA samples. Indeed, all the amplicons derived from this sequence clustered in one OTU at this cut-off.

Our tree-based treatment applied to NGS sequences demonstrated that few OTUs have been previously described by the traditional cloning-sequencing (CS) method. As these OTUs represent taxa present in relatively low abundance in many environments, little information is available about them. These novel OTUs were contained in a broad range of higher level taxa, including i) well-established clades such as Cryptomycota, ii) in phyla rarely detected by cultivation-independent sequencing (e.g., Ichthyosporea) and iii) in novel clades previously undescribed in lacustrine ecosystems, such as Foraminifera.

Thus, according to this study, the OTUs representing the most abundant sequences were found among Fungi, Alveolata, Stramenopiles, Cryptophyta and Rhizaria. More precisely, the phylogenetic affiliation allows to delineate three of the four previously defined freshwater Cryptophyta clades [6]. Within the Fungi, numerous OTUs were associated with Cryptomycota [57] or Chytridiomycota, which include both parasitic and saprotrophic organisms [58]. The presence of Chlorophyta and Haptophyta was confirmed in most of the lake environments sampled in this study. By the CS method used for describing PCC, Chlorophyta and Haptophyta were often absent [59], [60] or found at a very low proportion [6], [37], whereas these phyla represented a significant proportion of PCC when counting methods such as FISH were used [61]. Such a bias has also been highlighted in marine environments since epifluorescence microscopy reveals a dominance of phototrophic or mixotrophic cells over heterotrophic cells [62]. Another example of phyla rarely described yet detected here is the Ichthyosporea phylum, which was found only in hyper-eutrophic conditions [63]. Finally, some clades supported by high bootstrap values in our phylogenies, e.g., Mamiellales or Foraminifera, seem original because they have not been detected by CS with 'universal' eukaryotic primers. To our knowledge this is the first time that a clade closely associated to Mamiellales, as defined by Marin and Melkonian [64], has been detected in lakes. Present but scarce in our pyrosequencing data, these microalgae constitute the dominant photosynthetic group among the picoplankton 18S rRNA gene sequences in marine surveys (∼ 1/3 of the sequences), especially in coastal waters, and have been shown to account for 45% of the picoeukaryotic community, as targeted by TSA-FISH in these waters [65], [66]. The freshwater counterpart of this group, the Monomastigales, is rarely recovered from environmental samples and likely requires new molecular approaches that will specifically target photosynthetic organisms in the environment [64]. Freshwater Foraminifera, a group of granuloreticulosan protists largely neglected until now have already been detected by using specific primers in one study of freshwater ecosystems [67]. Thus, a NGS sequencing analysis with a moderate depth (∼ 10,000 cleaned read per sample for Eukaryota) allows for the detection of the main phylogenetic phyla but also rarely detected phyla or phyla only detected by specific primers which act similar to massively parallel sequencing by focusing on one clade. Among the biases commonly assigned to CS, other than the variability in the cell lysis efficiency, the rRNA gene copy number, which range from 1 to 12,000 [68] is certainly the most important and may result in an over-representation of heterotrophic organisms notably of the alveolate taxa [34]. However, even if these differences in copy number distort the interpretation in number of reads and OTUs for both the CS and NGS methods, the massively parallel sequencing can at least increase detection of rare lineages or organisms with low gene copy numbers thanks to the increased depth of sequencing. We can hypothesize that this copy number could be more homogeneous at a specific lower taxonomic level (for example Alveolata), and the various indexes were therefore computed for each phylum instead of considering the whole protistan community (Table S3).

## Conclusion

These results show that phylogenetic methods provide a clear edge in describing under-studied and complex communities, allowing the taxonomic affiliation of experimental sequences within an evolutionary framework; the study of relatedness among both environmental and reference sequences; and the evaluation of proximity of experimental sequences (“binning”). Thus, the tree-based method presented in this work, applied to the whole spectrum of microorganisms diversity (i.e., Eukaryota, Bacteria and Archaea), makes it possible to seek typical clades, allowing for the discovery of new putative lineages that are rarely or never recovered by classical sequencing approaches and the investigation of specific features within ecosystems considering sampling depths and periods. This feature cannot be inferred with a similarity search, a naïve Bayesian classification (RDP) or tree-based methods that process one sequence at a time.

## Materials and Methods

The data originating from simulations and pyrosequencing were processed by a pipeline, referred to as PANAM (Phylogenetic Analysis of Next-generation AMplicons) that is based on publicly available programs. In addition to the phylogenetic analysis, this pipeline allows for the complete analysis of a full pyrosequencing run, including raw data processing, sequence clustering into OTUs and generating phylogenies for the taxonomic affiliation. The description of the procedure is detailed in the following sections (*“Processing of raw pyrosequencing reads and OTU picking”; “Phylogenetic affiliation”; “Richness and diversity indexes”).* It is written in Perl and can be run on Linux. The package comprises a reference sequence database, a taxonomy file and reference profile alignments and can be obtained from http://code.google.com/p/panam-phylogenetic-annotation/.

### Processing of raw pyrosequencing reads and OTU picking

The pyrosequencing reads can be cleaned according to different methods commonly used in the field of molecular microbial ecology. Pyrosequencing errors can therefore be reduced by removing the primers (e.g., [69]), defining a minimal score and length of the reads (e.g., [14]) or removing reads with unidentified bases (Ns).

Short sequences and sequences with low-quality scores are removed using PANGEA scripts [16] and only sequences with a primer match percentage above a defined threshold are selected using Fuznuc [70]. Alternatively, other quality filtering methods can be implemented; the platform does not depend upon the filtering approach described above. When several samples are analyzed, the checked sequences are split into different files depending on their bar code or tag. Then, generated files are clustered using USEARCH [71] at a user-defined threshold, and representative sequences from OTUs are selected for the phylogenetic assignment.

### Phylogenetic affiliation

For the phylogenetic affiliation, a dedicated database of reference sequences, verified taxonomy and alignments was built using sequences extracted from the SSURef 108 database of the SILVA project [72]. For this purpose, all the sequences (16S and 18S rRNA genes) with more than 1,200 bp, quality score > 75%, and a pintail value > 50 were extracted. The sequence quality score defined by SILVA is a combination of the percentages of ambiguities, homopolymers longer than 4 bases and possible vector contaminations, and the pintail value corresponds to the probability that the rRNA sequence is chimeric. The complete database, after filtering according to the criteria above, contains 164,353 sequences (Archaea: 11,092; Bacteria: 131,428; and Eukaryota: 21,833) together with their taxonomy. To speed up the phylogenetic processing, the 3 domains were split into 37 phyletic groups of unicellular organisms corresponding to the first monophyletic clade after domains, as annotated in the guide-tree of SILVA (ARB format), and clustered at 97% identity.

Each profile corresponds to the first rank beneath that of domain. As the taxonomy of Bacteria and Archaea follow standardized taxonomic paths, the monophyletic profiles of these two domains correspond to phylum, the first level occurring after the domain. For Eukaryota domain, the taxonomy does not necessarily fit this organization, and the position of the taxon in the taxonomic hierarchy does not imply rank as it is the case with Bacteria and Archaea. Therefore, for the eukaryotic profiles, we opted for the rank position (the first one after the eukaryotic domain) and the monophyly, regardless to the taxonomic level.

For each of the 37 phyletic groups, an outgroup containing one sequence from each other group belonging to the same domain plus 2 external sequences were added to the alignment to root the phyletic tree to be produced and to specify the relatedness of early diverging sequences from the root of the group. To broaden the targeted diversity, the user can add specific environmental sequences to the database and the profiles.

**Figure 5:**
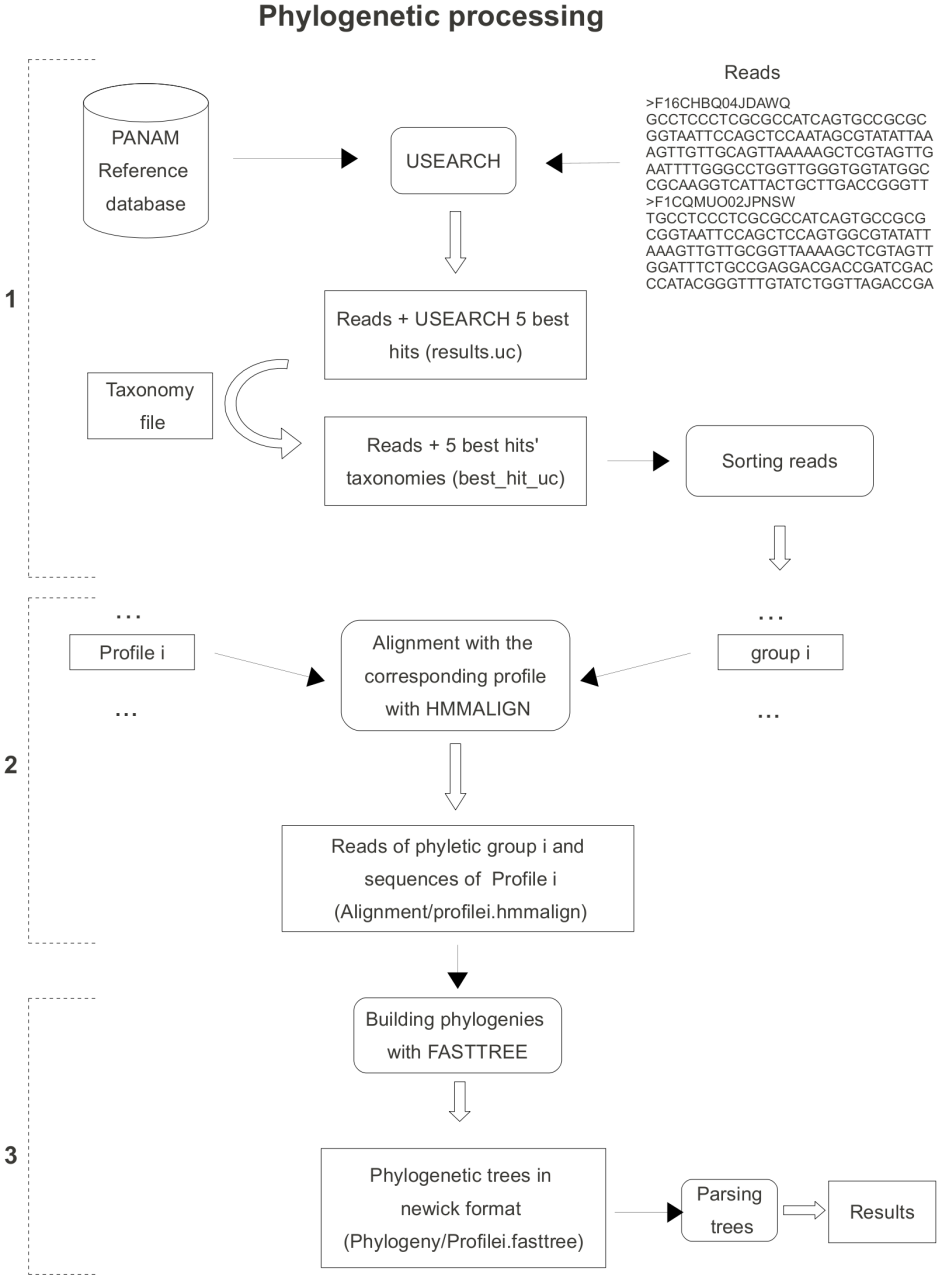
Flow chart describing the phylogenetic affiliation. A primary classification, sorts and splits reads into groups according to the taxonomy of their best USEARCH hit (1). Next, a file containing aligned reads and sequences from the corresponding group is generated by processing a profile alignment by HMMER. This file is used by FASTTREE to build a phylogenetic tree (2), which is then parsed to assign a taxonomy to each read and to report putative clades (3).

Using this dedicated database, the phylogenetic affiliation is carried out following the different stages described in the Figure 5.

1- First, OTUs are compared against the reference database described above with USEARCH [71]. As this first step does not intend to provide an exact affiliation, but rather to give a first approximation to perform a rapid and accurate phylogenetic analysis, the query sequences are sorted according to the taxonomy of their best hits, whatever their similarity score. Several files are generated, each containing the reads and their 5 best hits, assigned to one of the 37 specific phyletic groups.

2- After reads have been assigned to phyletic groups, they are aligned to the reference sequences of the corresponding profile alignment for that group using hmmalign from the HMMER package [73]. Synthetic files, which include the reference sequences and the aligned experimental reads, are generated.

3- Using FASTTREE [74], a bootstrapped phylogenetic tree (100 iterations) is built for each phyletic profile, including OTUs associated with their 5 best hits and the reference sequences. The trees are then parsed to generate files containing the taxonomy of the inserted sequences and files reporting the clades that could be identified from reads forming monophyletic groups. Two methods for taxonomy assessment are implemented: lowest common ancestor (LCA) and nearest neighbor (NN). In this last method, for each query sequence, all the nodes containing the sequence are scanned from the most recent to the deepest. The closest neighbor is defined as the first referenced sequence starting from the lowest node. The query sequence will acquire the complete taxonomy of its nearest neighbor. For LCA [32] each node holds only the common taxonomy between all of its descendants and thus may be incomplete. Each query sequence will inherit the taxonomy of its lowest node. The final taxonomy assignment is based on the phylogeny. The relatedness between all sequences (both experimental and referenced) are re-evaluated, and the similarity based assignments proposed on stage 1 are therefore revised to provide a more phylogeny-driven affiliation. Regarding the clades, their definition differs according to authors (e.g., [75], [76]), although in general, a new clade is declared when the cluster contains environmental sequences from at least 3 different sources and is supported by bootstrap values generally higher than 70%. The files generated describe monophyletic clusters with all the information required for experts in the field to define a putative environmental clade: a bootstrap value, a list of all the experimental sequences affiliated to it and the nearest reference neighbour together with its taxonomy. The implementation of PANAM (files generated) is extensively described in the documentation associated with the pipeline.

### Richness and diversity indexes

After the cleaning step, richness (Chao1 and ACE), diversity (Shannon) indexes, and coverage are computed for each sample [77]. Subsequently, sequence library sizes are equalized to avoid biases associated with different sampling depths (e.g., [78]). Briefly, the same number of sequences (i.e., the number of sequences in the smallest sample) are randomly sampled from each library, and diversity indexes are calculated for these equalized datasets. After phylogenetic affiliation, Chao1 and the Shannon diversity indexes are computed for levels 2 and 3 from the EMBL classification (e.g., Stramenopiles and Bacillarophyta ).

### Analysis of sequencing data obtained from simulations

PANAM was first tested on near full-length sequences with known taxonomy using 5 sets of 1000 sequences randomly picked from the reference database and removed from it for evaluations to be re-affiliated. The reliability of PANAM taxonomic affiliations was evaluated for specificity defined as the proportion of ranks correctly affiliated among the detected ones. A pyrosequencing simulation was also performed with pseudo-reads being generated by clipping the 5 × 1000 full-length sequences datasets from 6 universal forward primers for Eukaryotes [79] (Table S2). Clipped sequences were extended 200 and 400 bp from the forward primer positions defined on the *Saccharomyces cerevisiae* sequence (V01335), thus covering regions with different variability along the 18S rRNA gene. As emphasized, this pipeline allows taxonomic affiliations within an evolutionary context: its performance was thus primarily compared with that of STAP (Small Subunit rRNA Taxonomy and Alignment Pipeline) [51], the phylogenetic affiliation method used in WATERS (Workflow for the Alignment, Taxonomy, and Ecology of Ribosomal Sequences) [41], but was also compared with non-phylogenetic methods, including BLAST and the RDP Classifier implemented in MOTHUR [19] trained on the near full-length and trimmed sequences of the reference database.

The computational load of the phylogenetic analyses using PANAM was also tested with increasingly large datasets to evaluate processing time on a personal computer and to detect any scaling issues.

### Analysis of sequencing data obtained from environmental studies

The PANAM tree-based method was run on environmental sequences, namely i) a set of environmental sequences originating from published studies on the diversity of protists and belonging to described environmental lacustrine clades of Perkinsozoa and Cercozoa [6], [7] and ii) from an environmental survey of the lacustrine protist diversity performed in eight freshwater ecosystems.

For this purpose, eight lakes or reservoirs, described in Table 2 (Lakes Anterne, Aydat, Bourget, Godivelle, Geneva, and Pavin, and Reservoirs Sep and Villerest), were sampled once during their thermal stratification (from May to August according to the lake). Water samples from the epilimnion (1 to 5 m) were collected with a Van Dorn bottle at a permanent station (the deepest zone of the lake). Water samples (from 100 to 120 ml) were successively filtered through 5 µm-pore-size and 0.2 µm-pore-size polycarbonate filters (Millipore), and the membranes were stored at-80°C until nucleic acid extraction. All samples were extracted following the protocol described previously by Lefranc et al. [37].

The V4-V5 variable region of eukaryotic 18S rDNA was amplified with primers Ek-NSF573 and Ek-NSR1147 (Table S2). To discriminate each sample, a 5 bp multiplex tag was coupled with the Roche 454 pyrosequencing adaptor A. The amplification mix (30 µl) contained 30 ng of genomic DNA, 200 µM of deoxynucleoside triphosphate (Bioline, London, UK), 2 mM MgCl2 (Bioline), 10 pmol of each primer, 1.5 U of *Taq* DNA polymerase (Bioline) and the PCR buffer. The cycling conditions were an initial denaturation at 94°C for 10 min followed by 30 cycles of 94°C for 1 min, 57°C for 1 min, 72°C for 1 min and 30 s and a final 10-min extension at 72°C. Finally, the amplicons of all of the samples were pooled at equimolar concentrations and pyrosequenced using a Roche 454 GS-FLX system (Titanium Chemistry) by GATC (Konstanz, Germany). The reads, alignments and trees have been deposited in Dryad (http://datadryad.org). The reads used in this study were selected from a full run, separated into bins according to the tags, analyzed by PANAM, using trimming criteria of quality score > 22 and sequence length > 200 bases and clustering into OTUs with a 95% similarity threshold. UNIFRAC metrics [30] and a principal coordinate analysis were used to compare the small protist community between the lakes based on phylogenetic information obtained by PANAM using the packages Picante and ade4 implemented in the R software [80].

To broaden the covered diversity, more specifically regarding the environmental and pyrosequencing datasets processed in this study, and to build phylogenies with more similar sequences for the studied environment, 173 sequences from eukaryotic clades specific to lacustrine ecosystems, defined in previous works (e.g., [6], [37]), were introduced in the eukaryotic reference database and the corresponding groups.

## Supporting Information

Figure S1 **The Cercozoa (A) and Perkinsea (B) phylogenies generated by PANAM after inserting environmental sequences.** Inserted environmental sequences are in color (sequences with no accession number have been deposited in GenBank).
(PDF)

Figure S2 **Processing time of PANAM-LCA depending on the number and length of reads.**
(PDF)

Figure S3 **The Cryptomycota phylogeny displaying the representative OTUs detected in the lakes.** A representative OTU can be picked from a particular ecosystem but can be present in all ecosystems sampled as the OTU named Anterne08F F1CQMUO02ICISV.
(PDF)

Table S1 **Comparison of the different approaches of taxonomic assignment.**
(PDF)

Table S2 **The primers names and sequences used in the simulations and pyrosequencing.**
(PDF)

Table S3 **Main taxonomic groups with richness and diversity indexes in the different lakes studied.**
(PDF)
