# Supplementary material for: Phylogenetic Affiliation of SSU rRNA Genes Generated by Massively Parallel Sequencing: New Insights into the Freshwater Protist Diversity
Source: PLoS One. 2013 Mar 14;8(3):e58950. doi: 10.1371/journal.pone.0058950 (PMC3597552; doi:10.1371/journal.pone.0058950)
Supplement: Figure S1 — The Cercozoa (A) and Perkinsea (B) phylogenies generated by PANAM after inserting environmental sequences. Inserted environmental sequences are in color (sequences with no accession number have been deposited in GenBank). (PDF) [file pone.0058950.s001.pdf]

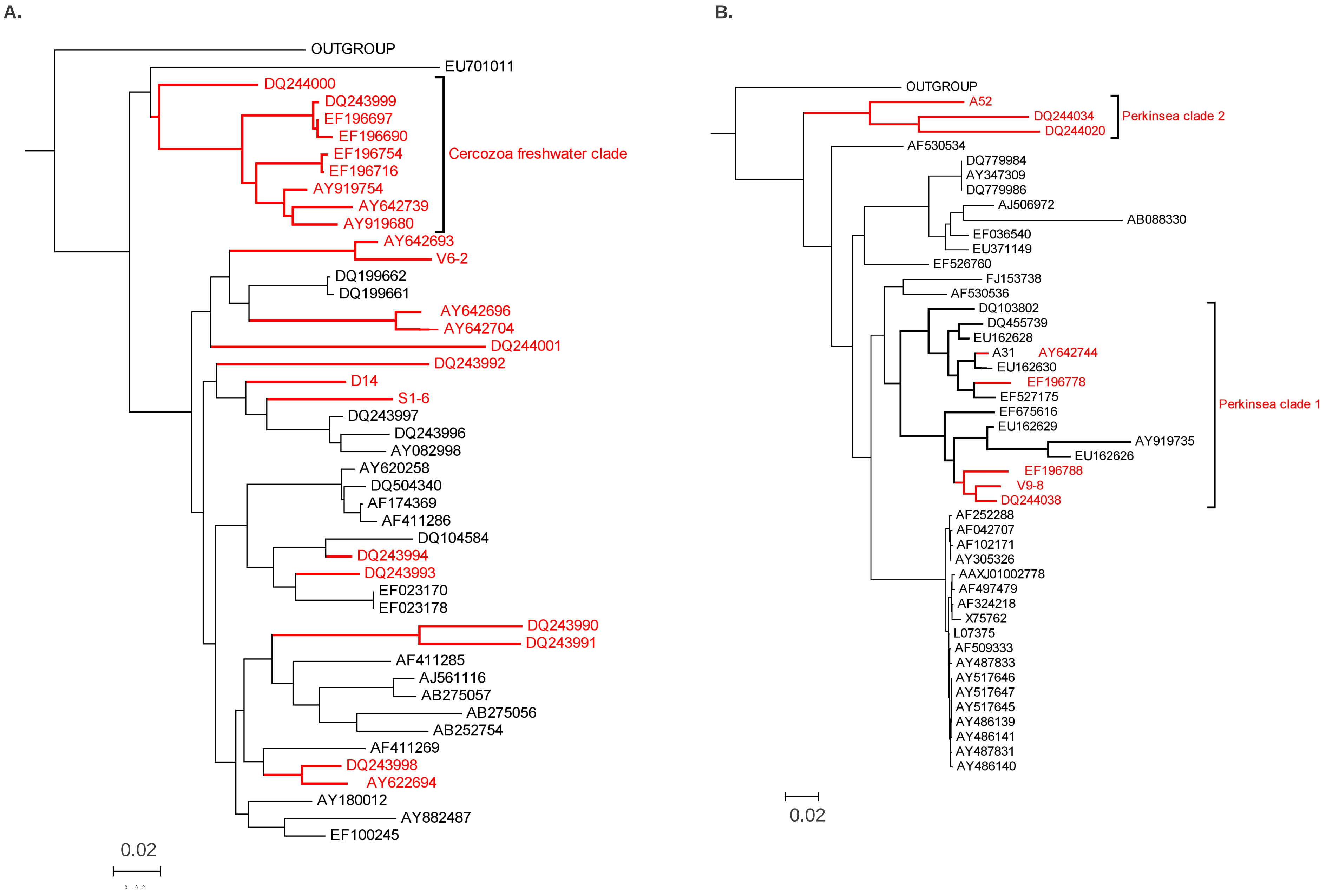

Fig. S1: The *Cercozoa* (A) and *Perkinsea* (B) phylogenies generated by PANAM after inserting environmental sequences. Inserted environmental sequences are in color (sequences with no accession number have been deposited in GenBank).
