# Supplementary figures and images for: Phylogenetic Affiliation of SSU rRNA Genes Generated by Massively Parallel Sequencing: New Insights into the Freshwater Protist Diversity
Source: PLoS One. 2013 Mar 14;8(3):e58950. doi: 10.1371/journal.pone.0058950 (PMC3597552; doi:10.1371/journal.pone.0058950)

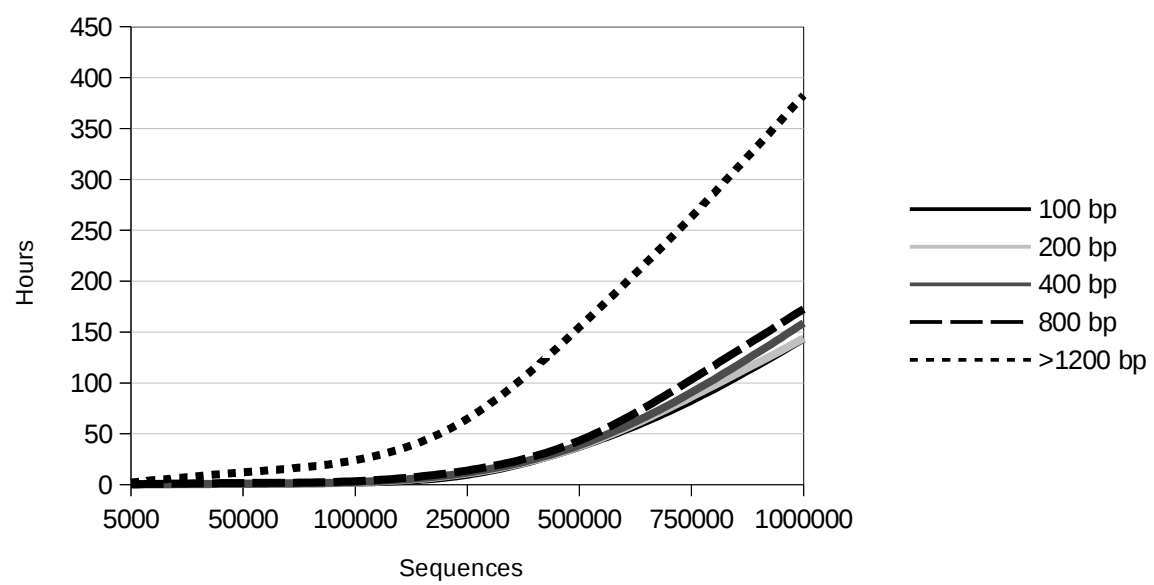

Fig. S2: Processing time of PANAM-LCA depending on the number and length of reads.

Supplement: Figure S2 — Processing time of PANAM-LCA depending on the number and length of reads. (PDF) [file pone.0058950.s002.pdf]
