# Supplementary material for: Phylogenetic Affiliation of SSU rRNA Genes Generated by Massively Parallel Sequencing: New Insights into the Freshwater Protist Diversity
Source: PLoS One. 2013 Mar 14;8(3):e58950. doi: 10.1371/journal.pone.0058950 (PMC3597552; doi:10.1371/journal.pone.0058950)
Supplement: Figure S3 — The Cryptomycota phylogeny displaying the representative OTUs detected in the lakes. A representative OTU can be picked from a particular ecosystem but can be present in all ecosystems sampled as the OTU named Anterne08F F1CQMUO02ICISV. (PDF) [file pone.0058950.s003.pdf]

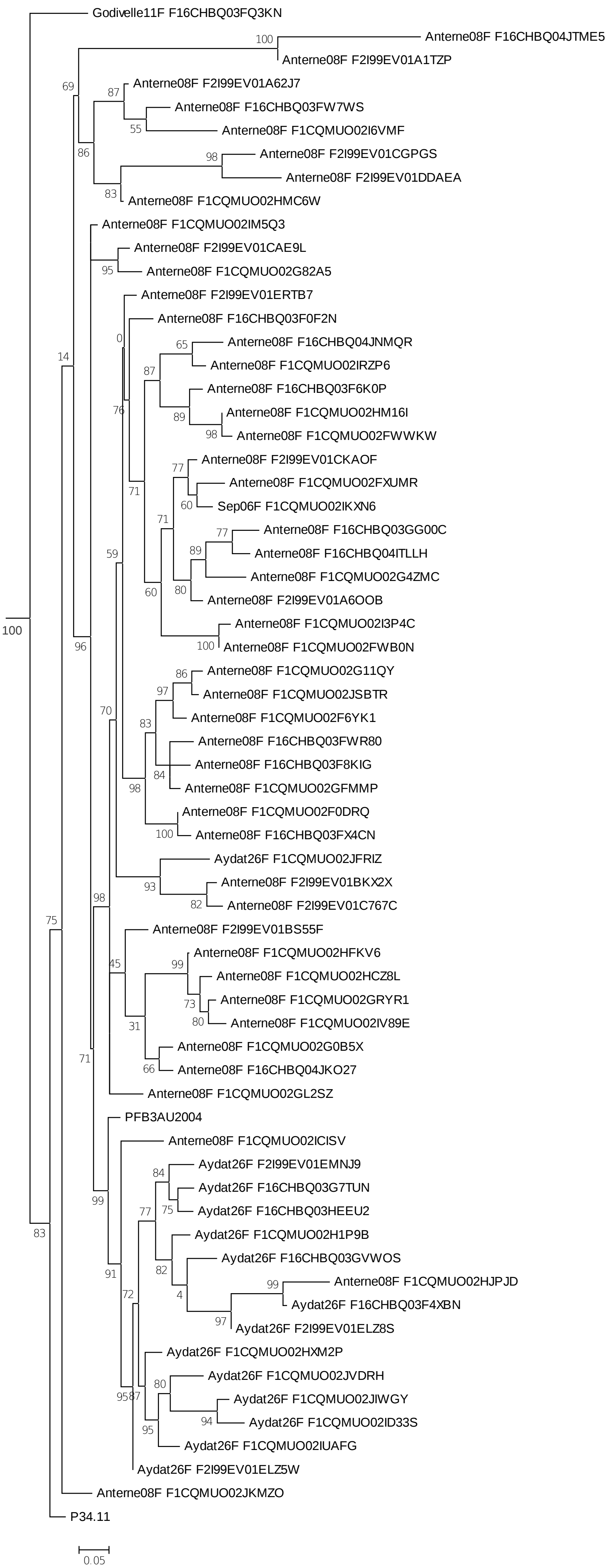

Fig. S3: The Cryptomycota phylogeny displaying the representative OTUs detected in the lakes. A representative OTU can be picked from a particular ecosystem but can be present in all ecosystems sampled as the OTU named Anterne08F F1CQMUO02ICISV.
