# Supplementary material for: Phylogenetic Affiliation of SSU rRNA Genes Generated by Massively Parallel Sequencing: New Insights into the Freshwater Protist Diversity
Source: PLoS One. 2013 Mar 14;8(3):e58950. doi: 10.1371/journal.pone.0058950 (PMC3597552; doi:10.1371/journal.pone.0058950)
Supplement: Table S1 — Comparison of the different approaches of taxonomic assignment. (PDF) [file pone.0058950.s004.pdf]

	Similarity search	Supervised classification	Tree-based methods
**Sequences**			
Minimal required length	~100 bp	~100 bp	~400 bp
Sensitivity to the region	+	+	++
**Database**			
Sensitivity to the size	++	+++	+
Sensitivity to the defined taxonomy	++	+++	-
Training	no	yes	no
**Processing needs**			
Time	++	+	+++
Ressources	+	+	+++
**Measure of relatedness**			
Taxonomy	E-values ; Similarity	Scores of confidence	Position on the phylogeny
Level of relatedness	The consensus taxonomy of top hits	The user defined score threshold	LCA
Description of clades	no	no	yes

Table S1 : Comparison of the different approaches of taxonomic assignment
