# Supplementary material for: Phylogenetic Affiliation of SSU rRNA Genes Generated by Massively Parallel Sequencing: New Insights into the Freshwater Protist Diversity
Source: PLoS One. 2013 Mar 14;8(3):e58950. doi: 10.1371/journal.pone.0058950 (PMC3597552; doi:10.1371/journal.pone.0058950)
Supplement: Table S2 — The primers names and sequences used in the simulations and pyrosequencing. (PDF) [file pone.0058950.s005.pdf]

Primer	Sequence
NSF4	CTGGTTGATYCTGCCAGT
NSF370	AGGGYTCGAYYCCGGAGA
NSF573	CGCGGTAATTCCAGCTCCA
NSF963	TTRATCAAGAACGAAAGT
NSF1179	AATTTGACTCAACACGGG
NSF1419	ATAACAGGTCTGTGATGCCC
NSR1147	CCGTCAATTYYTTTRAGTTT

Table S2: The primers names and sequences used in the simulations and pyrosequencing
