# Supplementary material for: Phylogenetic Affiliation of SSU rRNA Genes Generated by Massively Parallel Sequencing: New Insights into the Freshwater Protist Diversity
Source: PLoS One. 2013 Mar 14;8(3):e58950. doi: 10.1371/journal.pone.0058950 (PMC3597552; doi:10.1371/journal.pone.0058950)
Supplement: Table S3 — Main taxonomic groups with richness and diversity indexes in the different lakes studied. (PDF) [file pone.0058950.s006.pdf]

| | | Anterne | | | | | Aydat | | | | | Bourget | | | | | Godivelle | | | | |
|---|---|---|---|---|---|---|---|---|---|---|---|---|---|---|---|---|---|---|---|---|---|
| | | #seq | #OTUs | Schao1 | Shannon | Coverage | #seq | #OTUs | Schao1 | Shannon | Coverage | #seq | #OTUs | Schao1 | Shannon | Coverage | #seq | #OTUs | Schao1 | Shannon | Coverage |
| **Alveolata** | | 32 | 13 | 28.00 | 2.39 | 81.25 | 498 | 35 | 50.00 | 2.72 | 97.99 | 564 | 26 | 39.00 | 0.81 | 97.52 | 2450 | 50 | 69.12 | 1.92 | 99.27 |
| | Apicomplexa | 0 | 0 | | | | 38 | 2 | | | | 42 | 3 | | | | 12 | 5 | | | |
| | Ciliophora | 29 | 12 | | | | 245 | 20 | | | | 14 | 9 | | | | 2278 | 33 | | | |
| | Dinophyceae | 3 | 1 | | | | 183 | 11 | | | | 27 | 11 | | | | 149 | 10 | | | |
| | Perkinsea | 0 | 0 | | | | 32 | 2 | | | | 481 | 3 | | | | 11 | 2 | | | |
| **Amoebozoa** | | 0 | 0 | 0 | 0 | 0 | 0 | 0 | 0 | 0 | 0 | 0 | 0 | 0 | 0 | 0 | 0 | 0 | 0 | 0 | 0 |
| | Centramoebida | 0 | 0 | | | | 0 | 0 | | | | 0 | 0 | | | | 0 | 0 | | | |
| | Tubulinea | 0 | 0 | | | | 0 | 0 | | | | 0 | 0 | | | | 0 | 0 | | | |
| | unclassified Amoebozoa | 0 | 0 | | | | 0 | 0 | | | | 0 | 0 | | | | 0 | 0 | | | |
| **Choanoflagellida** | | 0 | 0 | | | | 2 | 1 | 1.00 | 0.00 | 100.00 | 0 | 0 | 0 | 0 | 0 | 0 | 0 | 0 | 0 | 0 |
| | unclassified Choanoflagellida | 0 | 0 | | | | 2 | 1 | | | | 0 | 0 | | | | 0 | 0 | | | |
| **Cryptophyta** | | 9 | 3 | 3.00 | 1.00 | 100.00 | 210 | 13 | 23.00 | 1.23 | 97.62 | 108 | 7 | 13.00 | 0.94 | 96.30 | 389 | 15 | 17.00 | 1.51 | 98.97 |
| | Cryptomonadaceae | 2 | 1 | | | | 11 | 2 | | | | 11 | 1 | | | | 207 | 4 | | | |
| | Cryptophyta nucleomorph | 0 | 0 | | | | 4 | 2 | | | | 1 | 1 | | | | 21 | 4 | | | |
| | Cryptophyta_2 | 5 | 1 | | | | 5 | 1 | | | | 0 | 0 | | | | 10 | 3 | | | |
| | Cryptophyta_3 | 2 | 1 | | | | 42 | 4 | | | | 95 | 4 | | | | 54 | 3 | | | |
| | Cryptophyta_4 | 0 | 0 | | | | 148 | 4 | | | | 1 | 1 | | | | 97 | 1 | | | |
| **Diplomonadida** | | 1 | 1 | 1.00 | 0.00 | 0.00 | 2 | 2 | 3.00 | 0.69 | 0.00 | 14 | 5 | 5.00 | 1.51 | 92.86 | 0 | 0 | 0 | 0 | 0 |
| | Hexamitidae | 1 | 1 | | | | 2 | 2 | | | | 14 | 5 | | | | 0 | 0 | | | |
| **Euglenozoa** | | 6 | 5 | 8.00 | 1.56 | 33.33 | 24 | 14 | 18.20 | 2.54 | 70.83 | 85 | 28 | 35.86 | 2.97 | 87.06 | 2 | 2 | 3.00 | 0.69 | 0.00 |
| | Euglenida | 6 | 5 | | | | 24 | 14 | | | | 85 | 28 | | | | 2 | 2 | | | |
| **Fungi** | | 16946 | 75 | 109.36 | 1.65 | 99.83 | 5604 | 49 | 68.12 | 0.70 | 99.68 | 613 | 69 | 115.87 | 2.06 | 93.80 | 704 | 63 | 84.08 | 2.34 | 96.73 |
| | Chytridiomycota | 0 | 0 | | | | 11 | 2 | | | | 3 | 2 | | | | 6 | 4 | | | |
| | Dikarya | 10 | 8 | | | | 27 | 14 | | | | 80 | 32 | | | | 80 | 22 | | | |
| | Glomeromycota | 0 | 0 | | | | 0 | 0 | | | | 0 | 0 | | | | 2 | 1 | | | |
| | LKM11 | 16893 | 56 | | | | 5411 | 20 | | | | 20 | 2 | | | | 27 | 5 | | | |
| | Microsporidia | 0 | 0 | | | | 0 | 0 | | | | 0 | 0 | | | | 2 | 1 | | | |
| | Rhizophydium clade | 1 | 1 | | | | 1 | 1 | | | | 0 | 0 | | | | 19 | 4 | | | |
| | Rozella clade | 0 | 0 | | | | 32 | 1 | | | | 0 | 0 | | | | 8 | 3 | | | |
| | Zygomycota_1 et rel. | 0 | 0 | | | | 12 | 1 | | | | 0 | 0 | | | | 3 | 1 | | | |
| | unclassified Fungi | 42 | 10 | | | | 110 | 10 | | | | 510 | 33 | | | | 557 | 22 | | | |
| **Haptophyceae** | | 0 | 0 | 0 | 0 | 0 | 23 | 1 | 1.00 | 0.00 | 100.00 | 24 | 1 | 1.00 | 0.00 | 100.00 | 3 | 2 | 2.00 | 0.64 | 66.67 |
| | Pavlovales | 0 | 0 | | | | 0 | 0 | | | | 0 | 0 | | | | 1 | 1 | | | |
| | unclassified Haptophyceae | 0 | 0 | | | | 23 | 1 | | | | 24 | 1 | | | | 2 | 1 | | | |
| **Heterolobosea** | | 2 | 1 | 1.00 | 0.00 | 100.00 | 0 | 0 | 0 | 0 | 0 | 0 | 0 | 0 | 0 | 0 | 0 | 0 | 0 | 0 | 0 |
| | Schizopyrenida | 2 | 1 | | | | 0 | 0 | | | | 0 | 0 | | | | 0 | 0 | | | |
| **Ichthyosporea** | | 0 | 0 | 0 | 0 | 0 | 0 | 0 | 0 | 0 | 0 | 18 | 2 | 2.00 | 0.45 | 100.00 | 2 | 1 | 1.00 | 0.00 | 100.00 |
| | unclassified Ichthyosporea | 0 | 0 | | | | 0 | 0 | | | | 18 | 2 | | | | 2 | 1 | | | |
| **Parabasalidea** | | 8 | 4 | 7.00 | 1.07 | 62.50 | 0 | 0 | 0 | 0 | 0 | 53 | 9 | 11.00 | 1.35 | 92.45 | 0 | 0 | 0 | 0 | 0 |
| | Trichomonada | 8 | 4 | | | | 0 | 0 | | | | 53 | 9 | | | | 0 | 0 | | | |
| **Rhizaria** | | 9 | 6 | 9.00 | 1.68 | 55.56 | 183 | 13 | 13.33 | 1.62 | 98.91 | 204 | 22 | 31.00 | 1.69 | 95.10 | 202 | 15 | 25.50 | 1.62 | 96.53 |
| | Cercozoa | 4 | 2 | | | | 173 | 10 | | | | 168 | 6 | | | | 201 | 14 | | | |
| | Foraminifera | 5 | 4 | | | | 10 | 3 | | | | 36 | 16 | | | | 1 | 1 | | | |
| **Rhodophyta** | | 1 | 1 | 1.00 | 0 | 0 | 1 | 1 | 1.00 | 0.00 | 0.00 | 23 | 7 | 10.00 | 1.57 | 87.00 | 0 | 0 | 0 | 0 | 0 |
| | Florideophyceae | 0 | 0 | | | | 1 | 1 | | | | 0 | 0 | | | | 0 | 0 | | | |
| | Bangiophyceae | 1 | 1 | | | | 0 | 0 | | | | 23 | 7 | | | | 0 | 0 | | | |
| **Viridiplantae** | | 4 | 4 | 7.00 | 1.10 | 0.00 | 260 | 16 | 18.00 | 1.80 | 98.46 | 1 | 1 | 1.00 | 0.00 | 0.00 | 235 | 12 | 14.00 | 1.56 | 98.71 |
| | Chlorophyta | 3 | 3 | | | | 259 | 15 | | | | 1 | 1 | | | | 233 | 10 | | | |
| | Streptophyta | 1 | 1 | | | | 1 | 1 | | | | 0 | 0 | | | | 2 | 2 | | | |
| **stramenopiles** | | 21 | 12 | 30.00 | 2.17 | 57.14 | 785 | 39 | 50.00 | 2.42 | 98.60 | 930 | 42 | 53.00 | 2.84 | 98.71 | 2700 | 83 | 86.93 | 3.36 | 99.59 |
| | Bacillariophyta | 7 | 1 | | | | 14 | 2 | | | | 0 | 0 | | | | 14 | 5 | | | |
| | Bicosoecida | 1 | 1 | | | | 1 | 1 | | | | 187 | 16 | | | | 364 | 21 | | | |
| | Chrysophyceae | 9 | 6 | | | | 221 | 16 | | | | 489 | 12 | | | | 1908 | 37 | | | |
| | Dictyochophyceae | 1 | 1 | | | | 32 | 6 | | | | 17 | 3 | | | | 280 | 9 | | | |
| | Eustigmatophyceae | 2 | 2 | | | | 270 | 2 | | | | 0 | 0 | | | | 0 | 0 | | | |
| | Labyrinthulida | 0 | 0 | | | | 15 | 3 | | | | 32 | 3 | | | | 55 | 4 | | | |
| | Oomycetes | 0 | 0 | | | | 91 | 3 | | | | 65 | 3 | | | | 3 | 1 | | | |
| | unclassified stramenopiles | 1 | 1 | | | | 141 | 6 | | | | 140 | 5 | | | | 76 | 6 | | | |

Tab. S3 : Main taxonomic groups with richness and diversity indices in the different lakes studied

| | | | | | Anterne | | | | | Aydat | | | | | Bourget | | | | | Godivelle | |
|---|---|---|---|---|---|---|---|---|---|---|---|---|---|---|---|---|---|---|---|---|---|
| | | #seq | #OTUs | Schao1 | Shannon | Coverage | #seq | #OTUs | Schao1 | Shannon | Coverage | #seq | #OTUs | Schao1 | Shannon | Coverage | #seq | #OTUs | Schao1 | Shannon | Coverage |
| **Alveolata** | | **32** | **13** | **28.00** | **2.39** | **81.25** | **498** | **35** | **50.00** | **2.72** | **97.99** | **564** | **26** | **39.00** | **0.81** | **97.52** | **2450** | **50** | **69.12** | **1.92** | **99.27** |
| | Apicomplexa | 0 | 0 | | | | 38 | 2 | | | | 42 | 3 | | | | 12 | 5 | | | |
| | Ciliophora | 29 | 12 | | | | 245 | 20 | | | | 14 | 9 | | | | 2278 | 33 | | | |
| | Dinophyceae | 3 | 1 | | | | 183 | 11 | | | | 27 | 11 | | | | 149 | 10 | | | |
| | Perkinsea | 0 | 0 | | | | 32 | 2 | | | | 481 | 3 | | | | 11 | 2 | | | |
| **Amoebozoa** | | **0** | **0** | **0** | **0** | **0** | **0** | **0** | **0** | **0** | **0** | **0** | **0** | **0** | **0** | **0** | **0** | **0** | **0** | **0** | **0** |
| | Centramoebida | 0 | 0 | | | | 0 | 0 | | | | 0 | 0 | | | | 0 | 0 | | | |
| | Tubulinea | 0 | 0 | | | | 0 | 0 | | | | 0 | 0 | | | | 0 | 0 | | | |
| | unclassified Amoebozoa | 0 | 0 | | | | 0 | 0 | | | | 0 | 0 | | | | 0 | 0 | | | |
| **Choanoflagellida** | | **0** | **0** | | | | **2** | **1** | **1.00** | **0.00** | **100.00** | **0** | **0** | **0** | **0** | **0** | **0** | **0** | **0** | **0** | **0** |
| | unclassified Choanoflagellida | 0 | 0 | | | | 2 | 1 | | | | 0 | 0 | | | | 0 | 0 | | | |
| **Cryptophyta** | | **9** | **3** | **3.00** | **1.00** | **100.00** | **210** | **13** | **23.00** | **1.23** | **97.62** | **108** | **7** | **13.00** | **0.94** | **96.30** | **389** | **15** | **17.00** | **1.51** | **98.97** |
| | Cryptomonadaceae | 2 | 1 | | | | 11 | 2 | | | | 11 | 1 | | | | 207 | 4 | | | |
| | Cryptophyta nucleomorph | 0 | 0 | | | | 4 | 2 | | | | 1 | 1 | | | | 21 | 4 | | | |
| | Cryptophyta_2 | 5 | 1 | | | | 5 | 1 | | | | 0 | 0 | | | | 10 | 3 | | | |
| | Cryptophyta_3 | 2 | 1 | | | | 42 | 4 | | | | 95 | 4 | | | | 54 | 3 | | | |
| | Cryptophyta_4 | 0 | 0 | | | | 148 | 4 | | | | 1 | 1 | | | | 97 | 1 | | | |
| **Diplomonadida** | | **1** | **1** | **1.00** | **0.00** | **0.00** | **2** | **2** | **3.00** | **0.69** | **0.00** | **14** | **5** | **5.00** | **1.51** | **92.86** | **0** | **0** | **0** | **0** | **0** |
| | Hexamitidae | 1 | 1 | | | | 2 | 2 | | | | 14 | 5 | | | | 0 | 0 | | | |
| **Euglenozoa** | | **6** | **5** | **8.00** | **1.56** | **33.33** | **24** | **14** | **18.20** | **2.54** | **70.83** | **85** | **28** | **35.86** | **2.97** | **87.06** | **2** | **2** | **3.00** | **0.69** | **0.00** |
| | Euglenida | 6 | 5 | | | | 24 | 14 | | | | 85 | 28 | | | | 2 | 2 | | | |
| **Fungi** | | **16946** | **75** | **109.36** | **1.65** | **99.83** | **5604** | **49** | **68.12** | **0.70** | **99.68** | **613** | **69** | **115.87** | **2.06** | **93.80** | **704** | **63** | **84.08** | **2.34** | **96.73** |
| | Chytridiomycota | 0 | 0 | | | | 11 | 2 | | | | 3 | 2 | | | | 6 | 4 | | | |
| | Dikarya | 10 | 8 | | | | 27 | 14 | | | | 80 | 32 | | | | 80 | 22 | | | |
| | Glomeromycota | 0 | 0 | | | | 0 | 0 | | | | 0 | 0 | | | | 2 | 1 | | | |
| | LKM11 | 16893 | 56 | | | | 5411 | 20 | | | | 20 | 2 | | | | 27 | 5 | | | |
| | Microsporidia | 0 | 0 | | | | 0 | 0 | | | | 0 | 0 | | | | 2 | 1 | | | |
| | Rhizophydium clade | 1 | 1 | | | | 1 | 1 | | | | 0 | 0 | | | | 19 | 4 | | | |
| | Rozella clade | 0 | 0 | | | | 32 | 1 | | | | 0 | 0 | | | | 8 | 3 | | | |
| | Zygomycota_1 et rel. | 0 | 0 | | | | 12 | 1 | | | | 0 | 0 | | | | 3 | 1 | | | |
| | unclassified Fungi | 42 | 10 | | | | 110 | 10 | | | | 510 | 33 | | | | 557 | 22 | | | |
| **Haptophyceae** | | **0** | **0** | **0** | **0** | **0** | **23** | **1** | **1.00** | **0.00** | **100.00** | **24** | **1** | **1.00** | **0.00** | **100.00** | **3** | **2** | **2.00** | **0.64** | **66.67** |
| | Pavlovales | 0 | 0 | | | | 0 | 0 | | | | 0 | 0 | | | | 1 | 1 | | | |
| | unclassified Haptophyceae | 0 | 0 | | | | 23 | 1 | | | | 24 | 1 | | | | 2 | 1 | | | |
| **Heterolobosea** | | **2** | **1** | **1.00** | **0.00** | **100.00** | **0** | **0** | **0** | **0** | **0** | **0** | **0** | **0** | **0** | **0** | **0** | **0** | **0** | **0** | **0** |
| | Schizopyrenida | 2 | 1 | | | | 0 | 0 | | | | 0 | 0 | | | | 0 | 0 | | | |
| **Ichthyosporea** | | **0** | **0** | **0** | **0** | **0** | **0** | **0** | **0** | **0** | **0** | **18** | **2** | **2.00** | **0.45** | **100.00** | **2** | **1** | **1.00** | **0.00** | **100.00** |
| | unclassified Ichthyosporea | 0 | 0 | | | | 0 | 0 | | | | 18 | 2 | | | | 2 | 1 | | | |
| **Parabasalidea** | | **8** | **4** | **7.00** | **1.07** | **62.50** | **0** | **0** | **0** | **0** | **0** | **53** | **9** | **11.00** | **1.35** | **92.45** | **0** | **0** | **0** | **0** | **0** |
| | Trichomonada | 8 | 4 | | | | 0 | 0 | | | | 53 | 9 | | | | 0 | 0 | | | |
| **Rhizaria** | | **9** | **6** | **9.00** | **1.68** | **55.56** | **183** | **13** | **13.33** | **1.62** | **98.91** | **204** | **22** | **31.00** | **1.69** | **95.10** | **202** | **15** | **25.50** | **1.62** | **96.53** |
| | Cercozoa | 4 | 2 | | | | 173 | 10 | | | | 168 | 6 | | | | 201 | 14 | | | |
| | Foraminifera | 5 | 4 | | | | 10 | 3 | | | | 36 | 16 | | | | 1 | 1 | | | |
| **Rhodophyta** | | **1** | **1** | **1.00** | **0** | **0** | **1** | **1** | **1.00** | **0.00** | **0.00** | **23** | **7** | **10.00** | **1.57** | **87.00** | **0** | **0** | **0** | **0** | **0** |
| | Florideophyceae | 0 | 0 | | | | 1 | 1 | | | | 0 | 0 | | | | 0 | 0 | | | |
| | Bangiophyceae | 1 | 1 | | | | 0 | 0 | | | | 23 | 7 | | | | 0 | 0 | | | |
| **Viridiplantae** | | **4** | **4** | **7.00** | **1.10** | **0.00** | **260** | **16** | **18.00** | **1.80** | **98.46** | **1** | **1** | **1.00** | **0.00** | **0.00** | **235** | **12** | **14.00** | **1.56** | **98.71** |
| | Chlorophyta | 3 | 3 | | | | 259 | 15 | | | | 1 | 1 | | | | 233 | 10 | | | |
| | Streptophyta | 1 | 1 | | | | 1 | 1 | | | | 0 | 0 | | | | 2 | 2 | | | |
| **stramenopiles** | | **21** | **12** | **30.00** | **2.17** | **57.14** | **785** | **39** | **50.00** | **2.42** | **98.60** | **930** | **42** | **53.00** | **2.84** | **98.71** | **2700** | **83** | **86.93** | **3.36** | **99.59** |
| | Bacillariophyta | 7 | 1 | | | | 14 | 2 | | | | 0 | 0 | | | | 14 | 5 | | | |
| | Bicosoecida | 1 | 1 | | | | 1 | 1 | | | | 187 | 16 | | | | 364 | 21 | | | |
| | Chrysophyceae | 9 | 6 | | | | 221 | 16 | | | | 489 | 12 | | | | 1908 | 37 | | | |
| | Dictyochophyceae | 1 | 1 | | | | 32 | 6 | | | | 17 | 3 | | | | 280 | 9 | | | |
| | Eustigmatophyceae | 2 | 2 | | | | 270 | 2 | | | | 0 | 0 | | | | 0 | 0 | | | |
| | Labyrinthulida | 0 | 0 | | | | 15 | 3 | | | | 32 | 3 | | | | 55 | 4 | | | |
| | Oomycetes | 0 | 0 | | | | 91 | 3 | | | | 65 | 3 | | | | 3 | 1 | | | |
| | unclassified stramenopiles | 1 | 1 | | | | 141 | 6 | | | | 140 | 5 | | | | 76 | 6 | | | |

Tab. S3 : Main taxonomic groups with richness and diversity indices in the different lakes studied
